# On moving targets and magic bullets: Can the UK lead the way with responsible data linkage for health research?

**DOI:** 10.1016/j.ijmedinf.2015.08.011

**Published:** 2015-11

**Authors:** G. Laurie, J. Ainsworth, J. Cunningham, C. Dobbs, K.H. Jones, D. Kalra, N.C. Lea, N. Sethi

**Affiliations:** a Mason Institute, School of Law, University of Edinburgh, UK; b Centre for Health Informatics, Institute of Population Health, University of Manchester, Manchester Academic Health Science Centre, Manchester, UK; c Swansea University Medical School, UK; d Centre for Health Informatics and Multiprofessional Education, University College London, UK

**Keywords:** Data linkage, Health research, Electronic health records, Information governance, Secondary uses

## Abstract

**Purpose:**

To provide an overview of essential elements of good governance of data linkage for health-related research, to consider lessons learned so far and to examine key factors currently impeding the delivery of good governance in this area. Given the considerable hurdles which must be overcome and the changing landscape of health research and data linkage, a principled, proportionate, risk-based approach to governance is advocated.

**Discussion:**

In light of the considerable value of data linkage to health and well-being, the United Kingdom aspires to design and deliver good governance in health-related research. A string of projects have been asking: what does good governance look like in data linkage for health research? It is argued here that considerable progress can and must be made in order to develop the UK’s contribution to future health and wealth economies, particularly in light of mis-start initiatives such as care.data in NHS England. Discussion centres around lessons learned from previous successful health research initiatives, identifying those governance mechanisms which are essential to achieving good governance.

**Conclusion:**

This article suggests that a crucial element in any step-increase of research capability will be the adoption of adaptive governance models. These must recognise a range of approaches to delivering safe and effective data linkage, while remaining responsive to public and research user expectations and needs as these shift and change with time and experience. The targets are multiple and constantly moving. There is not – nor should we seek – a single magic bullet in delivering good governance in health research.

## Highlights

• We explore key elements of good governance in health linkage.
• Adaptive reflexive governance models are essential.
• Two examples illustrate how we can achieve standardisation of practice.
• Distinct elements of governance compiled in a composite fashion tend to challenges.

## 1 Introduction: the problem and the vision

The recent debacle over care.data reveals yet another aspect of the multifaceted entity that is data sharing in healthcare [1]. The government and NHS England proposal to extract data from patient records for retention and use in a centralised database – with possible access from commercial entities – has not only generated considerable criticism, but led to suspension of the scheme, in order to allow better consultation with and involvement of patients and public [2]. The initiative has only very recently (partially) re-launched, and it remains to be seen how the four pathfinder projects progress and how they are received by the public [3]. Some of us have argued elsewhere that the initiative was premature and ill-conceived for want of ‘social licence’: that it, the false assumption that public confidence in GPs could simply be borrowed across to such an initiative [4]. This must also be set against the 2013 Caldicott 2 Review [5] into responsible sharing of patient data which, significantly, added a seventh principle to the Caldicott Guardians’ guiding principles: ‘[t]he duty to share information can be as important as the duty to protect patient confidentiality’ [6]. More recently, the Cabinet Office has published a discussion document on data-sharing policy. It points out that the common assumption that government departments can easily share data to improve services is false [7]. Against this, in turn, we have the on-going uncertainty over the legal position on data processing, driven by European Commission plans to introduce a Data Protection Regulation to tighten up the legal regimes across the continent [8], while in England the recent passing of the Care Act 2014 now gives power to the Health Research Authority (HRA) to authorise the processing of confidential medical information for medical research, subject to approval by an ethics committee (section 117), and requires the HRA to put ‘…in place and operate a system for reviewing decisions’ [9]. All of this typifies, and can be seen as a reaction to, a pre-existing problem identified by the Academy of Medical Sciences in a number of its outputs [10], namely, that a culture of caution prevails in data sharing for (health) research [11].

This is not to suggest that responsible research using health data cannot or does not happen. Indeed, the advent of the Farr Institute of Health Informatics Research builds on projects already delivered around the UK in each of the four nodes that make up the current consortium. Thus for example, SAIL [12]/CIPHER in Wales operates in a privacy-protecting safe haven [13]. There is a secure file transfer system in place for data being brought into the SAIL databank. Secure, remote data access is controlled and possible only when such access has been authorised. All output has to be approved. North of the border, the Scottish Health Informatics Programme (SHIP) has delivered a good governance framework to maximise the value of research using Scotland's rich health datasets. The framework is founded upon a mechanism of risk-based proportionate governance that reduces unnecessary regulatory burden without diluting appropriate scrutiny. At the University of Manchester and in collaboration with NHS partners, a technical solution to the ‘consent for consent’ problem was developed, enabling researchers to quickly and easily determine the likelihood of recruiting the required number of patients for a clinical trial protocol and to enact the recruitment process [14].

University College London (UCL) has developed an Identifiable Data Handling Service (IDHS) to allow authorised researchers to analyse clinical research data-sets within a data safe haven [15], where identifiable or pseudonymised data do not leave the secure boundary of the system. The service has also provided training workshops for researchers around information governance, and provides assistance for research projects when seeking both Information Toolkit Governance Level 2 compliance and exemption from the Common Law Duty of Confidentiality under Section 251 of the NHS Act.

The vision of the Farr initiative is:

‘To harness health data for patient and public benefit by setting the international standard for the safe and secure use of electronic patient records and other population-based datasets for research purposes’ [16].

The consortium comprises 24 academic Institutions and two Medical Research Council (MRC) units, bolstered by an additional £20 million in capital funds from the MRC. It aims to deliver high-quality, cutting-edge research linking electronic health data with both other forms of routinely collected data and other areas of research. It is also committed towards capacity building in health informatics research. The Farr Institute aims to provide the electronic infrastructure to facilitate collaboration across the four nodes, support their safe use of patient and research data for health and social care research. It will further enable partnerships through the provision of a physical structure which co-locates NHS organisations, industry, and other UK academic centres.

The common foundational principle that underpins all of the work of the Farr Institute is a commitment to responsible data sharing for the promotion of health and well-being. This commitment, in turn, is founded on a belief that scientifically sound, ethically robust data sharing for health research is in the public interest. This does not ignore the considerable importance of appropriate privacy and security measures, because – equally – robust protection of privacy is also in the public interest. However, nowhere is protection of privacy an absolute. This is true as much in law as in ethics. Indeed, the notion of absolute security of data is probably an unattainable goal, and certainly a foolish policy promise. No custodian of data should lead data subjects to believe otherwise. Responsible data management is about professional and responsible management of risk, and risk comes in many forms. It includes, but is not restricted to:

• invasion of privacy,
• potential discrimination or stigmatisation and resultant distress,
• economic threats and
• loss of trust.

In addition, any data custodian must consider risks to their reputational integrity if unjustifiable or irresponsible data linkages or disclosures are made. This is true even if such linkages or disclosures are entirely lawful. Good governance is not merely a matter of compliance with the law.

And yet the law poses considerable challenges for the data linkage aspirations of entities like the Farr Institute. Until now, the node activities have occurred in three distinct countries of the United Kingdom, subject to two different legal systems and over-shadowed by a European regime. The vision to lead international standards complicates matters further, especially any prospect of international data travel. Any attempt to harmonise national – let alone international – arrangements would be futile. There can be no one-size-fits-all approach to such rich and complex regulatory settings. Rather, the governance approach of the Farr Institute is considerably more realistic – to bring about mutual recognition of standards and best practices, drawing on lessons to date from regional successes, and considering where common ground and approaches might be extrapolated to other environments. Approximation is key.

As a crucial first step in this process of approximation of standards, the Farr governance team has identified critical areas of attention which serve as the foundational elements of good governance frameworks, and thus the starting points for further deliberation and construction of initiatives on a larger and more publicly-valuable scale. It is important to stress that the ethos is one of *co-production* of good governance between data custodians, potential data users, and data subjects themselves through robust and iterative engagement. It also requires transparent development and equitable access policies. The immediate lessons from care.data include the serious inadequacy of assuming that it is sufficient to attempt to inform data subjects unidirectionally through leafleting alone. Effective communication with stakeholders, especially with those for whom privacy is in play, must go beyond the mere provision of information. Equally, it is not enough simply to pass law. Care.data had a legal basis under the Health and Social Care Act 2012 [17], but this still did not prevent the adverse reaction to what was proposed. Although the government has attempted to provide yet further legal clarity in the Care Act 2014 for the processing of confidential medical information under the auspices of the Health Research Authority [18], the law can do no more than lay out broad legal parameters for operation. The real challenges will be in the Act's implementation through transparent responsible practices. This is where the approach of the Farr Institute can offer important insights.

## 2 The approach

The Farr initiative has drawn on its cumulative research expertise thus far to reveal the following features that, we suggest, must necessarily form part of any good governance framework.

### 2.1 Consent: the stalwart panacea?

One of the enduring features of discussions about what constitutes good governance in data linkage for research is the question of the role of individual consent. In other contexts, such as those involving research with patients' bodies or their tissues, the need for consent is rarely questioned. As a mechanism to give expression to individual wishes and to give effect to individual self-rule (autonomy), consent is seen as a self-evident truth and non-negotiable regulatory measure of respect [19]. There has, accordingly, been considerable borrowing of value from these other contexts to make similar claims about the crucial need for consent in information governance. But the settings are not the same.

When stripped of identifiers and other personalised characteristics, and processed at an aggregate level, it is not clear that it remains meaningful for any of us to talk of ‘my data’. Also, consent can give an illusion of control and security, when in fact, the only real power that it confers is an ability to say No. And, even if an individual says No, other factors might be in play that justify processing of data, such as significant public interests or even the vital interests of the individual him- or herself. For this and other reasons, consent is neither necessary nor sufficient for legitimate and lawful data protection. It is true that, if given, consent can provide a lawful basis for processing data [20], But, it is not strictly needed. Finally, consent suffers from a practical limitation in that it is almost always an up-front, one-off event [21]. While a ‘right to withdraw’ can, of course, exist through the life-course of a project, this casts consent (or, rather, refusal) as an extreme response. As such, consent is ill-equipped to ensure that appropriate two-way communication and robust oversight of research projects delivers on all interests that are in play.

While acknowledging the considerable value that is imbued in consent, the Farr Institute asks whether it is appropriate to cast consent as the driving governance mechanism. This is not to reject a role for consent altogether, but rather to see consent as one part of a cascade of governance mechanisms that can be deployed *after* a robust assessment of what is at stake in any proposed linkage scenario. This has not been the general approach to date.

### 2.2 Anonymisation: the tower of babel?

The mantra of ‘*consent or anonymise*’ was the rule of thumb that the former Patient Information Advisory Group (PIAG) used during 2002–2004 to explain the legislation, guidelines and medical professionals’ duties of confidence to the research world regarding the use of identifiable information outside of the care setting [22]. As information has become more widely used for research purposes, the appreciation of what *consent* and *anonymise* mean has evolved, but the rule of thumb has endured. Both are separate and distinct: the latter is a means of stripping out identifying attributes from datasets so that they can be used legally for purposes beyond which they were originally collected; the former is an affirmative action that individuals can use to express their autonomy. It is the most obvious basis of expressing trust when individuals share sensitive information about themselves. Anonymisation, on the other hand, is used to mitigate risks of identifying individuals, protect their rights to privacy and to deliver the medical profession's duty of confidentiality. It does not necessarily require consent to perform these roles. However in the process, anonymisation also often renders datasets less useful, because moderately identifying data are key to answering certain research questions: their removal makes linking separate datasets harder, and potentially, research findings less robust, and potentially useless. This has resulted in both legislators and data custodians trying to establish a balanced approach, whereby bona fide research uses are not unnecessarily hindered, do not break the law, nor do they deny a role for consent where this is thought to be necessary [23]. What is less widely acknowledged is the nature of anonymisation techniques: none of these can guarantee anonymity and current accepted practice takes a pragmatic view over whether the risk of re-identification is so low that it can be discounted. For example, the Information Commissioner’s Office issued guidance in 2012 that takes such a pragmatic approach. It recommends deploying a ‘motivated intruder’ test to assess whether anonymisation techniques are sufficiently robust to allow release or use of data. This asks whether ‘…a person who starts without any prior knowledge but who wishes to identify the individual from whose personal data the anonymised data has been derived’ is reasonably likely to achieve re-identification [24]? Another pertinent rule of thumb is that there is always a risk of error and/or accidental disclosure, and the counteractive techniques will only be as good as the people who develop and apply them. Periodic review of techniques and procedures is crucial to good governance. We cannot assume that data adequately anonymised today will always remain so.

By the same token, the goals of initiatives like the Farr Institute depend on being able to reliably link (when appropriate permissions are granted) records between research data-sets – a practice rendered extremely challenging after anonymisation techniques are applied. This prompted the practice of replacing identifiable attributes with a pseudonym, referred to as pseudonymisation. This process provides a means of not only matching more reliably between datasets, but also a link back to the identifiable record for those who are authorised to do so. Obviously this raises the likelihood of unauthorised participant re-identification during the practice of research, placing a greater duty of care on data controllers, a need for more robust risk mitigation strategies and rendering anonymisation redundant in some cases.

### 2.3 Consent or anonymise: challenging the existing paradigm

Despite its immediate and superficial appeal, the limitations of the ‘consent or anonymise’ paradigm have been well recognised in research governance circles. The establishment of the PIAG in 2001 is precisely an example of how such limitations have been addressed. The group acted in an advisory capacity to the Secretary of State to authorise uses of patient-identifiable data for research when consent was neither present nor practicable [25]. It has since morphed into the Confidentiality Advisory Group (CAG) acting under the HRA [26], and it is the HRA that now has the legal power to authorise data linkages for research under the new Care Act 2014. This, however, must be subject to research committee approval. Thus the role of CAG – or a similar entity – is set to become a permanent centre stage feature in the research governance arena. As a governance approach, it is an example of ‘authorisation’ of research practices. Namely permissions on linkage occur only after careful and close deliberation of the merits and risks of any proposed research using data. The PIAG and CAG have gone to great lengths to be transparent about their processes [27]. A similar approach was adopted in Scotland through the Privacy Advisory Committee (PAC), established in 1991, to advise two of the largest custodians of heath data in the country: National Services Scotland of NHS Scotland and the National Registers of Scotland [28]. It operated on similar levels of transparency and accountability. The PAC approvals process has recently been superseded by the newly formed Public Benefit and Privacy Panel for Health and Social Care (PBPP) [29]. The PBPP provides a streamlined approvals process for applications wishing to access NHS Scotland-originating data. Again, the PBPP is dedicated to facilitating robust and transparent scrutiny of data access, and the Panel bolsters direct involvement of members of the public in both scrutinizing and deciding upon data access applications. However, a notable difference north and south of the border is the role of legislation. Scotland has not seen the need to legislate, relying instead on the public interest in promoting scientifically sound, ethically robust research. These are the guiding parameters for the work of the PBPP. In England and Wales, the legislative route has been preferred, and we see the same guiding principles now enshrined in the Care Act 2014 which requires the HRA to:

‘…have regard to the need—

(a) To protect participants and potential participants in health or social care research and the general public by encouraging research that is safe and ethical, and
(b) To promote the interests of those participants and potential participants and the general public by facilitating the conduct of such research.’ (Section 111(2))

Furthermore, Section 111(3) of the 2014 Act states that:

‘The HRA must promote the co-ordination and standardisation of practice in the United Kingdom relating to the regulation of health and social care research; and it must, in doing so, seek to ensure that such regulation is *proportionate*.’ [30] (emphasis added).

No further specifics on how to discharge these duties are provided. We suggest that it is here that the work of the Farr Institute can prove to be invaluable. We offer two examples from our work to date: (i) the development and operation of the safe haven in Wales, and (ii) the design and delivery of proportionate governance in Scotland.

### 2.4 The safe haven: a new panacea?

A safe haven can be defined as a specialist, well governed, independently scrutinised [31] and accredited environment. There are different models within the concept of the safe haven, with some also serving long-term repositories for a collection of datasets as well as hosting an environment where data can be accessed for research. This section uses the Secure Anonymised Information Linkage (SAIL) system as a case study to outline briefly the role of the safe haven, including some of its main strengths and weaknesses. The SAIL system is an example of a safe haven that is also a repository, and it contains a range of anonymised routine datasets about the population of Wales.

Subject to regulatory and governance approvals, anonymously linked data required to answer research questions are made accessible within the safe haven, which is referred to as the SAIL Gateway. The Gateway acts as an analysis platform with a range of software packages, so that data can be analysed therein without being released from the system. One of the main strengths of a system such as SAIL is in having the data in one databank, thus saving time in data acquisition and facilitating research readiness. However, this may also be perceived by some as a weakness, and a risk as a possible Big Brother [32]. Thus it is imperative that (i) such systems have robust data security and governance frameworks, (ii) are subject to independent scrutiny and (iii) proposals to use the data are assessed to ensure they have the potential for public/patient benefit whilst safeguarding privacy. Even so, there are many multi-faceted challenges in striking the balance between data security and usefulness for research, requiring considerable investment in infrastructure and expertise. Although they appear to be emerging as the ‘new panacea’, safe havens may not provide the solution in all cases. Some organisations may not wish to or may not be allowed to by law to export their data. Furthermore, public(s) assurance and confidence are paramount to ensuring acceptability.

### 2.5 Principled, proportionate, and risk-based governance

The Scottish Health Informatics Programme (SHIP) ran from 2009 to 2013 and was funded by the Wellcome Trust to facilitate so-called secondary uses of heath data for research, building on the long-established, high-quality datasets held within Scotland [33]. Working closely with NHS Scotland as the principal data custodians of much of Scotland's health data, the SHIP interdisciplinary consortium identified a set of key barriers to more effective and efficient sharing. These were: (i) lack of clarity of what was permitted by law, (ii) confusion about when actors had the legal responsibility of acting as ‘data custodian’, (iii) lack of streamlined processes for data linkage approvals (and agreed parameters for doing so), and (iv) unmet need in information governance training [34]. The result was the development and delivery of a Good Governance Framework (GGF) for SHIP, consisting of four elements:

1 An account of responsibilities of key actors and decision-makers (largely a matter of clarifying who is a data controller).
2 A capacity building facility for researcher training and accreditation and wider awareness-raising (delivered through distance learning).
3 A statement of Principles and Best Practices to guide decision-making.
4 A mechanism of principled proportionate governance in making data linkage assessments.

Importantly, the GGF embraces a principles-based approach, that is, it recognises that sensitive decisions must be made that require careful exercises of judgment [35]. Accordingly, it does not seek to be overly prescriptive through a set of hard and fast rules, but rather, provides decision-makers with key principles to consider when scrutinising data linkage requests. Key principles include the need to demonstrate public interest in the proposed data linkage, and to require applicants to recognise and minimise privacy risks. A crucial additional pragmatic component of the GGF is its mechanisms of considering what proportionate governance looks like. It asks three fundamental questions: is the linkage request dealing with,

1 safe people, e.g. those accredited by SHIP?,
2 safe data, e.g. those linked through SHIP, and
3 safe environments, e.g. a SHIP safe haven or equivalent, such as SAIL [36].

If the answer to all of these questions is Yes, then a fast-track route that requires no further scruinty of the application is available for researchers. If the answer to any of these questions is No, then other more scrutiny-intensive routes must be taken, including full review by the PBPP. This, then, is an example of proportionality in action, and one from which the HRA might learn some useful lessons.

### 2.6 What has public engagement told us?

The lessons from the public and professional responses to the initial roll-out of care.data are sobering. Any public interest or good public-based initiative must first have a clear basis for making a claim that its foundations are well-established. As indicated above, care.data did have a legal basis in the Health and Social Care Act 2012, but this was not enough on its own to prevent a backlash. Law alone is insufficient to command public and stakeholder support. Moreover, law works far better when it puts in place barriers or protections against action; it is less adept at promoting particular ends that require sensitive and sensible human exercises of judgment. Additionally, any (legal) framework for decision-making on data linkage must demonstrate its ability to remain fit for purpose over time, and to continue to reflect public groups’ and other stakeholders’ expectations, which can and do change. Thus for care.data, it was not enough to say that similar types of use and linkage were already practised, albeit on a far less ambitious scale. Finally and crucially, the prospect of profit clearly impacts on what different sectors of society will tolerate, both within a public health service and with respect to private and confidential data [37].

Here again lessons can be learned from the work of the Farr Institute. Many of our regional efforts to date have involved public and stakeholder engagement, and our approaches to governance have taken the results into account. For example, in SHIP our focus group work showed a strong and repeated preference for a role for consent in data linkage decisions, albeit that, when explored further, most participants recognised and accepted practical limitations of consent for some kinds of research (as outlined above) [38]. Notwithstanding and as a direct response, the SHIP Good Governance Framework now provides that any application to use data through SHIP must address directly the question of whether a consent-based approach could be used. While it is possible to argue that anonymisation or authorisation are more appropriate routes for any given application, all applicants must reflect on the consent route and provide robust reasons as to why it should not be followed. On commercial involvement, our team's earlier public engagement work on the Generation Scotland project – to build a data and tissue resource to explore the role of genes and environment in the onset of disease – showed that there are multiple ‘publics’ with differing views [39]. Specific patient groups, for example, can tend to be more tolerant of commerce than health groups [40]. Most interestingly and discerningly, however, our research found that it is not so much the prospect of profit that publics find unacceptable, but the idea of ‘obscene’ profit. Moreover, there is some evidence – to be tested further – that commitments to benefit sharing can address some of these concerns [41].

The work of Farr CIPHER in Wales is built upon the governance principles developed in the SAIL system [42], and there is an active Consumer Panel for Data Linkage Research [43,44] that, in dialogue with various SAIL panels, boards etc.; provides a public viewpoint on the re‑use of data in research. The Panel expressed a strong desire to be better informed about the data linkage studies taking place, recognising that often academic proposals and outputs are written for experts in the field. As a result, the Information Governance Approval Form now includes not only a section for a lay summary, but also a section on the public engagement strategy. Further, and as anchored in the Consumer Panel’s Strategic Plan, strategies and measures are now in place to facilitate and audit its increasing input into data linkage studies.

Such transparency is reflected in the practices of CAG and the PBPP with respect to their approvals. Additonally, the Care Act 2014 now provides that the HRA must publish guidance on ‘… (a) principles of good practice in the management and conduct of health and social care research; [and] (b) requirements, whether imposed by enactments or otherwise, to which persons conducting health or social care research are subject.’

Most recently, the Farr Institute held a workshop with data custodians and researchers in order to identify key barriers against and facilitators of data reuse for health research purposes [45]. The lack of transparency around care.data and the resultant damage this may have caused in terms of eroding trust around data reuse emerged as a key concern amongst participants. Civic engagement as a means of engendering transparency as well as raising awareness around the public benefits of data reuse in research were key priorities for stakeholders. A recent UK data sharing review echoes these sentiments. The report stresses that building trust ‘is the responsibility of every digital stakeholder in the UK. It is a joint effort, which needs to be a focus for the Government, business, academia and the public’ [46].

Additional key findings suggest that risk aversion is a significant hindrance to data sharing and it was suggested that this was caused by misunderstandings around which data reuses are legally permitted and prohibited (and under which conditions). In particular, different interpretations arose between data custodians and researchers around consent requirements. These key concerns will inform the future work of the Farr Institute and will shape governance approaches developed throughout the course of the initiative.

### 2.7 Constantly moving targets: cross-sectoral and international linkages

All of the above advances have been achieved in the health sector and within individual countries of the United Kingdom. The Farr Institute is designed to build on these developments at the UK level, but the real step-changes that will signal success will be demonstrated by delivering on these standards beyond the Farr community in the UK. Two key targets are cross-sectoral linkages, and safe and effective linkages beyond Farr

At present, a parallel initiative funded by the ESRC is its Administrative Data Linkage Centres (ADRC) Network [47], with an associated linkage service. The service is designed to facilitate research based on linked, routinely-collected administrative data – for example education, crime, housing and employment – whilst also including health data. There are particular challenges in accessing and sharing some administrative datasets. For example, HM Revenue and Customs is currently prevented by law from releasing certain datasets to third parties such as data linkage centres or researchers [48]. Innovative methods will be needed to enable safe and meaningful data re-use. To this end, the Administrative Data Taskforce reported in December 2012 [49], and the government responded in June 2013 [50]. The Taskforce’s principal recommendation was the establishment of ADRCs in each of the four countries in the UK, as is now the case. Further and in its endorsement of this, the government pointed to the good practices emerging from SHIP and SAIL, and indicated that these had been strengthened ‘…from April 2013 when the Health and Social Care Information Centre (HSCIC) gained increased powers to provide data linkage services that can be used by others [51].’ This, of course, is the body which has been wrangling with the fall-out out from care.data. Notwithstanding and as pointed out above, the salutary lesson is that mere legislative provision to facilitate data linkage and sharing is not enough. Indeed, this raises further questions about another of the core recommendations of the Taskforce: the need for legislation. On this point the government was more circumspect, calling first for a mapping exercise to identify precisely (i) the current barriers and (ii) where existing powers might be sufficient but under-used. This reflects the work of the Farr Institute thus far, in that our research revealed not so much a problem with law per se, but a prevailing attitude in some quarters that militated against data use. Such a cultural reluctance cannot merely be legislated away. The proof of the benefits of sharing and linking must be clearly, amply and repeatedly demonstrated to increase the public mandate. Indeed, this goal was identified as a key priority by Farr workshop participants.

A final and crucial recommendation from the Taskforce was also recognised by the government: the need for a strategy for engaging the public. However, we would caution against the tone of the government’s response in terms of what can be reasonably expected from any such strategy or plan of engagement. To cite the governmental reply: ‘The public must be confident that access will only be made available for legitimate research purposes to approved researchers and that the outputs from analysis will in no way compromise their privacy [52].’

This sets the bar very high in terms of privacy protection. If it is to suggest that a ‘no risk’ approach to privacy is the only appropriate standard, it is likely to set up the entire enterprise for failure. This is not to suggest that the highest standards should not be sought, but rather to say that realistic expectations must be laid down. All data use and linkage comes with some degree of risk. This is simply a reality. Governance mechanisms such as safe havens, accreditation, risk-based proportionate governance and improved researcher training all do considerable work to minimise those risks, as well as to maximise likely benefits. But it is foolhardy to suggest that a zero tolerance approach to privacy is possible. It is likely only to result in disappointment and the undermining of trust.

### 2.8 Realising the vision

The vision of the Farr Institute is to harness health data for patient and public benefit by setting the international standard in trustworthy reuse of electronic patient records and related linkable data for large-scale research.

When dealing with multiple sectors, diverse stakeholders, divergent legal systems and myriad datasets, it is clear that no single model of governance will be suitable in all situations. The strength of the Farr Institute is that it demonstrates the value of distinct elements of governance that can be compiled in a composite fashion to deliver the most robust framework in any given context, while remaining flexible and adaptable over time. The emerging top-level message is that no single paradigm nor governance tool is necessarily a preferred option. ‘Consent or anonymise’ has been shown to be inadequate on its own, and has necessitated a third option of ‘authorisation’. Safe havens are important technical means to deliver high levels of security, but they do not provide answers on the logically-prior ethical judgments about whether data access should be granted or certain data linked.

Other quasi-technical solutions are being developed elsewhere that attempt to empower citizens throughout the research process, such as the notion of *dynamic* consent whereby preferences (or opt-outs) can be exercised as a research project evolves [53]. The important point to note about these efforts is the value-base that they reflect. This is the notion of the primacy of the individual over other considerations such as the public benefit of robust research. Added to this, there might be unintended privacy consequences of such approaches, because they will necessarily require a technical ability to always link downstream data uses to an identifiable individual, in order for future preferences to be traced and given effect. Thus while dynamic consent offers the prospect of continuing control that can be delivered by technical means, an on-going commitment to consent as a driver of health research governance privileges particular values and interests over others, here notably individualistic concerns over wider public interests.

The Farr Institute does not envisage any one governance tool as necessarily optimal. Rather, we would emphasise the importance of the triangulation of good governance, whereby consent, anonymisation, and authorisation can work together and be deployed in different measures depending on the particular circumstances of any proposed linkage. It should not be forgotten, as so often happens, that even an authorisation body can require specific, informed consent to be obtained if it believes that this is the best means to deliver the overall set of interests at stake.

Transparency in all of these efforts must be re-emphasised, with respect to decisions about linkages and with respect to access policies and practices equally. This can be achieved in large part through publication on websites and social media, but the passive provision of one-way information is only part of the picture. As care.data has shown, the reality is that many citizens will remain in the dark about many data linkage initiatives. Thus a robust and sustained strategy of genuine public engagement is required, such as that recommended by the ESRC’s Administrative Data Taskforce.

As for the research community itself, there must be a clear and open invitation to access data safely, with appropriate safeguards. Further, it will be crucial for all parties to understand that, in advocating ‘open access’, there is no endorsement whatsoever of data as a free-for-all. In other words, not everyone should have access to all data available, regardless of their motivation or ability to meet the necessary ethical and legal standards. Rather, the Farr governance team endorses the approach of the Royal Society in its 2012 report – Science as an Open Enterprise [54] – which helpfully proposes a model of ‘Intelligent Openness’. This mandates robust curation of valuable data, openness to sharing and clear commitment to appropriate protection of privacy. Among other things, it promotes principles not only of accessibility, but also of verifiability and of intelligibility. This last criterion must be true as much for citizens as for researchers who would access data.

There are many examples that suggest that the legislative route is of limited value, especially with respect to promoting trust in data linkages. Law must, of course, set non-negotiable parameters of what is clearly unacceptable. Beyond this, however, good governance must support and assist data linkage decision-makers to weigh up a range of considerations and exercise good judgment. Good governance rests unavoidably upon discretion; considerations change over time and are another example of the moving targets in this article. Good governance shifts with these changes, and delivers understanding and reassurance of roles, responsibilities and abilities with respect to responsible data sharing and linkage.

Finally, more needs to be done to deliver genuine engagement with stakeholders and public groups. We still have a lot to learn about what that means. It should include, for example, the possibility of influencing matters, including the direction of research where appropriate. Two-way communication is key, as reflected from the recent Farr workshop where participants stressed the need for meaningful civic engagement. This is not just about delivering one-way information about what is going on. Moreover, it must reflect a humility that we do not know all the answers before we begin. This emphasises, once again, the crucial importance of adaptive and receptive governance mechanisms.

## 3 Conclusion

The Farr Institute is aligning practices across institutions and the countries of the UK with seamless data sharing for multiple purposes. The common ethos is an openness to mutual learning and cooperation. In delivering on this, we suggest that the following key lessons must be taken on board:

1. There is no magic bullet in delivering good governance: a variety of approaches is required; identifying and adopting these approaches is crucial for the UK.
2. Guiding principles of responsible data linkage are a defensible, flexible and adaptable approach that can be of value across sectors and countries. Central among these are the importance of proportionality and mutual recognition of what counts as good practice.
3. Stakeholder and public engagement is key both to inform and to shape governance over time – models must be receptive and adaptive; the worth and the sustainability of any approach will stand or fall by its ability to demonstrate these features.

Whilst the UK still has considerable progress to make, it is taking considerable steps towards delivering upon its goals for data linkage.

**Summary table**

The authors shall provide a table with in 2–4 bullets statements on ‘what was already known on the topic’ and also in 2–4 bullets statements on ‘what this study added to our knowledge’. Note that the second part of the table should not list the results of the study as such. It should address what this study has proven and what insights have been gained.

What is already known on the topic?

• Data linkage for health-related research is a growing and complicated regulatory area.
• Currently, many valuable uses of health data for health research are being impeded.
• A standardisation of practice is sought around how data uses are governed, whilst at the same time ensuring proportionate regulation.
• Consent, anonymisation and most recently, safe havens have been considered as solutions to the myriad governance challenges which regulation of health data face.

What this study has added to our knowledge?

• Key elements of good governance.
• Adaptive and reflexive governance models are essential in order to achieve good governance and to address future challenges.
• Two concrete examples are offered illustrating how we can achieve standardisation of practice which facilitates proportionate governance.
• Distinct elements of governance which are compiled in a composite fashion can tend to current and future challenges.

**Competing interests**

We wish to confirm that there are no known conflicts of interest associated with this publication.

**Author declaration**

We confirm that the manuscript has been read and approved by all named authors and that there are no other persons who satisfied the criteria for authorship but are not listed. We further confirm that the order of authors listed in the manuscript has been approved by all of us.

We confirm that we have given due consideration to the protection of intellectual property associated with this work and that there are no impediments to publication, including the timing of publication, with respect to intellectual property. In so doing we confirm that we have followed the regulations of our institutions concerning intellectual property.

We understand that the Corresponding Author is the sole contact for the Editorial process (including Editorial Manager and direct communications with the office). He/she is responsible for communicating with the other authors about progress, submissions of revisions and final approval of proofs. We confirm that we have provided a current, correct email address which is accessible by the Corresponding Author and which has been configured to accept email from (nayha.sethi@ed.ac.uk)

Contributions of each author towards FARR Project and towards this article
